# Procainamide for the Rapid Suppression of Premature Ventricular Contractions: An (Almost) Forgotten Tool in the Cardiologist’s Armamentarium

**DOI:** 10.3390/diagnostics11020357

**Published:** 2021-02-20

**Authors:** Daniele Muser, Pasquale Santangeli, Gaetano Nucifora

**Affiliations:** 1 Cardiac Electrophysiology, Cardiovascular Medicine Division, Hospital of the University of Pennsylvania, Philadelphia, PA 3400, USA; daniele.muser@gmail.com (D.M.); pasquale.santangeli@pennmedicine.upenn.edu (P.S.); 2 Cardiac Imaging Unit, NorthWest Heart Centre, Manchester University NHS Foundation Trust, Manchester M23 9LT, UK

In the last few years, cardiac magnetic resonance (CMR) imaging has progressively acquired a central role in the diagnosis and management of patients with ventricular arrhythmias (VA). In particular, due to its tissue characterization capabilities almost resembling an “in vivo” histological examination, it has demonstrated the ability to identify subtle structural myocardial abnormalities missed by routine diagnostic investigations, including ECG and echocardiography, and potentially explains the origin of VA [1,2,3]. In patients presenting with VA and normal left ventricular (LV) function, the detection of underlying structural heart disease, frequently in the form of non-ischemic LV scar, has been associated with a high risk of life-threatening VA during follow-up, deserving proper medical attention, further risk stratification and careful evaluation for primary prevention implantable cardioverter defibrillator (ICD) [2,3,4].

Unfortunately, performing CMR in patients with repetitive VA poses several challenges related to the variability in the RR cycle. Usually, image acquisition is synchronized to the patient’s heart rhythm in order to always acquire data at the same selected time point of the cardiac cycle for a given image. In this context, the occurrence of premature beats determines data acquisition at different time points, resulting in significant blurring of the final CMR image [5]. Several methods have been developed to overcome these limitations, including the use of a prespecified image acquisition window within the RR interval and arrhythmia rejection algorithms. However, since the acquisition window is usually set at the patient’s shortest expected RR interval, it does not cover the complete cardiac cycle, resulting in the end-diastole not being completely sampled as well as in the prolonged-acquisition and breath-holding time [6]. Another possibility relies on real-time “single-shot” prospective triggering, in which the entire image is acquired in a single heartbeat; this approach, however, results in suboptimal image quality compared to segmented acquisitions [7].

So far, most CMR studies enrolling patients with frequent premature ventricular contractions (PVCs) have treated patients with oral antiarrhythmic drugs (AADs) for at least one week before CMR examination in order to suppress/reduce the PVC burden and optimize ECG trigger and image acquisition [1,2,3]. However, this cannot always be possible, especially in large volume centers with patients being referred from outside facilities. Moreover, the efficacy of oral AAD treatment is suboptimal, with only a mild reduction in the PVC burden in most of the cases [8,9].

In this issue of Diagnostics, Nikolaidou et al. move our knowledge a step forward by exploring the possibility to administer AAD intravenously at the time of CMR evaluation [10]. The authors have prospectively tested the efficacy and safety of intravenous administration of procainamide during the CMR scan in a group of 50 patients (mean age 48 ± 16 years; 52% female) referred to CMR for frequent PVCs (88% of the cases) and/or non-sustained ventricular tachycardia (12% of the cases). The vast majority of patients (84%) had an unremarkable previous medical history and echocardiographic findings, while only 8% of them had a mild to moderate reduction of LV function. Procainamide was administered within the scanner with intermittent intravenous boluses of 50 mg every minute until the complete suppression of PVCs up to a maximum dose of 10 mg/kg during continuous ECG monitoring and non-invasive blood pressure monitoring every minute. Overall, PVCs were completely suppressed in 20 (40%) patients and a significant reduction (≤1 PVC every 10 beats) was obtained in another 21 (42%) patients with an average dose of procainamide of 567 ± 197 mg over 11 ± 4 min. This determined a significant improvement of image quality from an average quality score of 1.62 ± 0.49 to 3.46 ± 0.51 (*p* < 0.001) on a 4-point scale. An uneventful but still significant drop in blood pressure (from a mean of 133/68 mmHg to a mean of 121/64 mmHg) was observed after procainamide administration, while there were no changes in heart rate, QRS duration, and QT duration. Of note, intravenous administration of procainamide effectively suppressed VA, even in patients already treated with oral AAD (beta-blockers in 73% of the cases, flecainide in 7% and amiodarone in another 7%).

Procainamide is a class IC agent no longer widely used as unavailable in most countries, which acts directly as fast sodium channel blocker and as potassium channel blocker through its active metabolite N-acetylprocainamide. So far, only two small, randomized trials have analyzed its role in VT treatment [11,12]. In both studies, procainamide was used intravenously for the acute termination of monomorphic-tolerated VT. Gorgels et al. demonstrated the superiority of procainamide at an infusion rate of 100mg/min in terminating VT in 80% of patients compared to 21% of those treated with intravenous lidocaine while in the PROCAMIO trial procainamide 10 mg/kg/20 min was demonstrated to be safer (9% vs. 41% of adverse events) and more effective (67% of acute VT termination vs. 38%) compared to intravenous amiodarone [11,12]. Potential side effects include QT prolongation and, most importantly, hypotension, which may be encountered in up to 30% of the cases [11,12,13]

Overall, the results of the study by Nikolaidou et al. are compelling and offer additional evidence supporting the efficacy and safety of the intravenous use of procainamide for VA suppression. However, we feel that this suggested approach should be carefully considered and tailored upon single patient characteristics, as further prospective investigations in larger series of patients are warranted. In this regard, it is important to highlight that pro-arrhythmic effects have been reported in up to seven percent of the patients treated with AAD for VA with the higher incidence in patients with severely reduced LVEF [14]. In general, the choice of a particular drug and its dose should be based on its efficacy in controlling VA and potential pro-arrhythmic effects as well as other side effects. Only a minority of patients with mild-to-moderate LV dysfunction were enrolled in this study, and the reported analyses focused on patients with no previous medical history, thereby precluding the widespread application of these findings on the whole population of “all-comers” presenting with repetitive non-sustained VA.

Recently, new image acquisition techniques such as accelerated real-time cine imaging with compressed sensing, real-time temporal parallel acquisition, and motion-corrected free-breathing averaged LGE imaging have been proposed to obtain good-quality images in patients with irregular rhythm and difficulties in breath holding [15,16,17]. However, those techniques were not implemented in the present study as they were not available at the investigating center. Further investigations comparing the use of AAD vs. state-of-the-art acquisition techniques are warranted as they may allow the obtainment of images of good enough quality to avoid the administration of potentially harmful AAD.

Another notable result of the study was that CMR abnormalities were documented in 58% of the cases despite 84% of the patients having negative echocardiographic findings; this confirms the greater sensitivity of CMR compared to echocardiography in the diagnosis of subtle myocardial structural abnormalities [3,18].

Despite the limitations related to the small sample size and the study design, the investigation by Nikolaidou et al. provides important incremental knowledge on the efficacy and safety of “on-demand” intravenous AAD utilization and suggests a role for procainamide to acutely suppress frequent VA in patients undergoing CMR evaluation, which warrants further investigation with larger prospective studies on a more heterogeneous population, including patients with systolic dysfunction and several cardiac disorders such as ischemic heart disease, primitive arrhythmic disorders, and non-ischemic cardiomyopathies. Based on this study, the use of intravenous procainamide seems to be safe and highly effective in temporarily suppressing VA, at least in patients with normal LV function and no major comorbidities, significantly improving CMR image quality.
